# Biomechanics of medial meniscus tears in the context of pain: a finite element analysis

**DOI:** 10.3389/fbioe.2025.1693500

**Published:** 2026-01-09

**Authors:** Karol Daszkiewicz, Piotr Łuczkiewicz

**Affiliations:** 1 Department of Mechanics of Materials and Structures, Faculty of Civil and Environmental Engineering, Gdańsk University of Technology, Gdańsk, Poland; 2 II Clinic of Orthopaedics and Kinetic Organ Traumatology, Medical University of Gdansk, Gdańsk, Poland

**Keywords:** finite element, knee, medial meniscus, meniscal tear, nociceptors, pain

## Abstract

**Introduction:**

Meniscal tears represent the most common knee pathology and may be associated with pain. Meniscal pain is caused by direct mechanical stimulation of nociceptors located in the outer, vascular part of the meniscus. Due to difficulties in selecting the optimal surgical treatment, the aim of the study was to analyze the influence of meniscal tears on the stress state in the context of meniscal pain.

**Methods:**

Radial, oblique, longitudinal and horizontal tears involving up to 90% of the width of the medial meniscus were modelled using the finite element method. Two types of knee joint loading were simulated: the stance phase of the gait cycle and external tibial rotation combined with compression.

**Results:**

The highest Tresca equivalent stresses were obtained for the radial tear of the posterior horn. The largest increase in mean shear stress (187%) on the outer surface of the meniscus, relative to the intact meniscus model, was observed for the oblique tear. Neither complete nor partial horizontal tears were associated with changes in shear stress in the innervated part of the meniscus.

**Discussion:**

Increased shear stresses in the innervated part of the meniscus, which may result in pain, were obtained in radial and oblique meniscal tears models. In the longitudinal tear model, instability of the inner part of the meniscus and increased shear stress were observed in the central part of the meniscus and at both ends of the tear.

## Introduction

Meniscal tears represent the most common knee pathology and are known to cause knee joint pain (Dandy, 1990; Kamimura et al., 2015). In most such cases, the recommended surgical treatment is excision of the unstable meniscus tissue or meniscal reconstruction (Abram et al., 2020). The partial meniscectomy has become one of the most frequently performed orthopedic procedures (Järvinen and Guyatt, 2016). Taking into account that meniscal tears are common in patients without any clinical symptoms the controversy arise regarding the choice of optimal therapeutic methods (Englund et al., 2008). Several studies have demonstrated that meniscal tears are associated with pain (Bin et al., 2008; Kamimura et al., 2015), while others have shown no relationship between meniscal lesions and clinical symptoms (Bhattacharyya et al., 2003; Englund et al., 2007). The neural element responsible for the development of meniscus pain are nociceptors, i.e., specialized peripheral neurons capable of receiving and transmitting signals following tissue damage (Assimakopoulos et al., 1992; Mine et al., 2000). They are located in the vascular outer third of the meniscus, known as the red zone, and in the horns near the meniscal roots (Lin et al., 2019; Orellana et al., 2024). According to the most popular „meniscal pain theory”, meniscal pain is caused by direct mechanical stimulation of nociceptors in the innervated part of the meniscus or their indirect stimulation from traction on the capsule in the case of instability of the torn meniscus (Mine et al., 2000). Mechanical nociceptors respond to cell deformation by activating transient receptor potential channels (Delmas et al., 2011).

Previous finite element studies have mainly investigated the influence of radial tears on the stress distribution in articular cartilage and meniscus, the contact area between meniscus and cartilage, and meniscus extrusion (Bedi et al., 2010; Łuczkiewicz et al., 2015; Zhang et al., 2019; 2025; Steineman et al., 2022; Wang et al., 2022; Yang et al., 2025). Among other types of meniscal tears, the following have been analyzed in a few studies: oblique tear (Li et al., 2020), longitudinal tear (Kedgley et al., 2019; Sukopp et al., 2024) and horizontal cleavage tear (Chen et al., 2023). These studies also investigated the effect of partial meniscectomy and suture meniscal repair on the peak contact pressure on the cartilage and the contact area.

To the best of our knowledge, there are no studies that have analyzed the biomechanics of meniscal tears, taking into account their potential influence on nociceptor activation. In clinical studies, it is usually difficult to assess the relationship between meniscal tear and the occurrence of pain. This is due to the presence of additional pathological changes in the knee joint, which may be as important a cause of pain as meniscal tissue damage (Farina et al., 2021). The aim of the study is to analyze the influence of most common types of meniscal tears on the stress state in the innervated zone of the medial meniscus and the risk of meniscal pain.

## Materials and methods

### Finite element models

**FIGURE 1:**
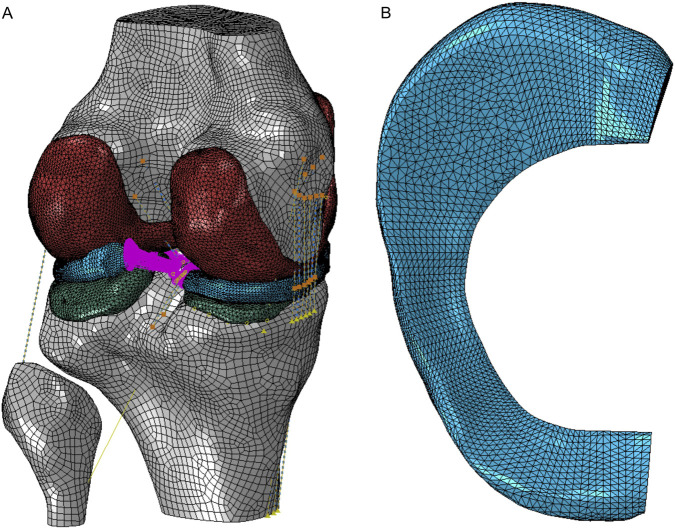
Finite element model of the intact knee joint **(A)**; finite element mesh of the medial meniscus **(B)**.

The three dimensional geometry of the knee joint was created in Mimics software (Materialise NV, Leuven, Belgium) based on magnetic resonance imaging scans of the left knee of a healthy female volunteer (43-year old). Finite element (FE) model of the intact knee joint was developed in Abaqus 6.14–2 (Dassault Systemes Simulia Corp., Providence, RI, USA) and described in detail in the previous paper (Daszkiewicz and Łuczkiewicz, 2021). The model consisted of menisci, articular cartilage (tibial, femoral), bones (tibia, fibula, femur) and ligaments (Figure 1A).

In finite element method (FEM) simulations, bones were modelled as rigid bodies and their kinematics was described by the translational and rotational degrees of freedom of their reference points (RPs). Two node three dimensional axial connector elements CONN3D2 were used to model ligaments and specific ligament bundles. Two bundles were defined for anterior cruciate ligament (ACL) and posterior cruciate ligament (PCL), and three bundles for medial collateral ligament (MCL) to better describe the anatomical role of ligaments. A single bundle was used for the lateral collateral ligament (LCL), anterolateral ligament (ALL) and posterior oblique ligament (POL). The meniscotibial ligament (MTL), meniscofemoral ligament (MFL) and anterior intermeniscal ligament (AIML) were modelled by the six CONN3D2 elements to reduce stress concentrations at their attachment points to the menisci.

**FIGURE 2:**
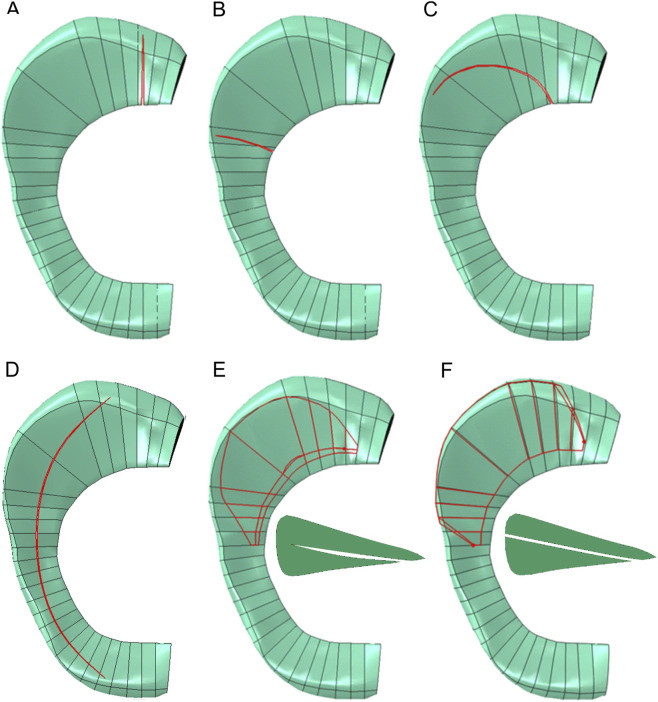
Types of meniscal tears. **(A)** Radial tear of the posterior horn. **(B)** Radial tear of the middle body. **(C)** Oblique tear. **(D)** Longitudinal tear. **(E)** Partial horizontal tear. **(F)** Full horizontal tear.

Additional six finite element models were developed in this study to investigate the effects of radial, oblique, longitudinal and horizontal medial meniscal tears on the stress state in the medial meniscus. The geometry of meniscal tears was assumed based on the medial meniscus tear morphology (Awh, 2008; Kamimura et al., 2015; Jarraya et al., 2017). Considering, that Bedi et al. (2010) showed that only large radial tears involving 90% of the width of the medial meniscus had impact on the stress distribution and the knee biomechanics, only 90% radial tears were analyzed in the current study. The models with the partial radial tear of the posterior horn and middle body are shown in Figures 2A,B, respectively. The oblique tear was introduced into the model as a vertical tear originating from the inner edge of the posterior horn extending up to 90% of the width of the meniscus (Figure 2C). A longitudinal tear, running parallel to the meniscal fibers, was created between the posterior and anterior horns of the meniscus (Figure 2D). Partial (up to 90% of the width) and full horizontal tears were modelled in the posterior part of the meniscal body and in posterior horn of the medial meniscus (Figures 2E,F).

First order tetrahedral elements (C3D4) of average size of 0.8 mm and 1.1 mm were used in discretization of menisci (Figure 1B) and articular cartilage, respectively. The FE mesh of intact knee model consisted of 268,634 tetrahedral finite elements (Figure 1A). The assumed discretization for all FEM models was positively verified in the mesh convergence study. The validation of the finite element model was described in paper (Daszkiewicz and Łuczkiewicz, 2021).

### Materials

The intact and torn menisci were modelled using an elastic and transversely isotropic material. A significantly greater Young’s modulus *E*_θ_ = 120 MPa was assumed in the circumferential direction of the fibers than in the cross-sectional plane *E*_p_ = 20 MPa. The values of the shear modulus *G* = 57.7 MPa and Poisson’s ratios *v*_θp_ = 0.3, *v*_p_ = 0.2 were taken from literature (Lechner et al., 2000; Haut Donahue et al., 2003; Yang et al., 2010). The tension only springs were used to model meniscal horn attachments. The total tensile stiffness of 253.5, 106.6, 218.0 and 218.7 [N/mm] was assumed for lateral anterior, lateral posterior, medial anterior and medial posterior horn attachment, respectively (Hauch et al., 2010; Daszkiewicz and Łuczkiewicz, 2021).

The strain energy potential of the Yeoh model was used to describe nearly incompressible, hyperelastic behavior of the articular cartilage. The nonzero parameters *C*_10_ = 2.0 MPa, *C*_20_ = 4.5 MPa, *D*_1_ = 0.0517 MPa^-1^ were assumed for the tibial articular cartilage and *C*_10_ = 1.4 MPa, *C*_20_ = 3.6 MPa, *D*_1_ = 0.0739 MPa^-1^ for the femoral cartilage (Robinson et al., 2016). The stiffness and the initial strain for specific ligaments and ligament bundles modelled by CONN3D2 elements were given in Table A in Supplementary Material. The density of 1,500 kg/m^3^ and 2,000 kg/m^3^ was assumed for soft tissues and bones, respectively (Erdemir and Sibole, 2010; Maharaj et al., 2013).

### Finite element analysis

Two types of knee joint loading at which patients experience meniscus pain were simulated: the stance phase of the gait cycle and external tibial rotation combined with a compressive force. Walking is one of the most common causes of the medial meniscus posterior root tear (Furumatsu et al., 2019). While external tibial torsion under load is used in Thessaly test to induce pain and assess the knee for medial meniscus tears (Blyth et al., 2015). In the initial static step, the equilibrium state was achieved between the contacting surfaces of articular cartilage and meniscus for the assumed initial strains in the ligaments. In order to improve the convergence of nonlinear computations in this step, automatic stabilization with a factor of 0.2 was applied in the contact formulation. The classical Lagrange multiplier method was used to enforce interaction between contacting surfaces in normal direction and the penalty method with small friction coefficient of 0.02 was used to describe tangential behaviour (Warnecke et al., 2019).

An implicit dynamic analysis was performed to simulate two loading scenarios. In the gait analysis, initial loads, moments and flexion angle were introduced into the model within one second (Daszkiewicz and Łuczkiewicz, 2021). Then, the stance phase lasting 0.6404 s was simulated, during which the standardized average forces and moments (Bergmann, 2008; Bergmann et al., 2014) were applied to the tibia RP. These loads were recalculated based on the volunteer body weight of 68 kg and transformed from an implant-based coordinate system to a knee joint coordinate system (Daszkiewicz and Łuczkiewicz, 2021). In the second loading scenario, a compressive force of 667 N corresponding to the body weight was applied to the tibia RP in the first second of the dynamic analysis and then a 15-degree external rotation of the tibia relative to the femur was imposed in the second s of the simulation.

All rotational and translational degrees of freedom (DOFs) of femur were fixed during the analysis. Flexion rotation of the tibia was prescribed in the gait analysis (Bergmann et al., 2014) and fixed in the external tibial rotation simulation. The remaining degrees of freedom of the tibia RP were not restricted. The inner cartilage surfaces were rigidly connected to the bones using a “tie'' constraint. Kinematic coupling between the fibula RP and the tibia RP was defined to constrain motion between these bones.

Gong et al. (2022) showed that shear stress, not stretch, plays a critical role in nociceptor activation. Since the nociceptor detect excessive stress and their activation leads to the experience of meniscal pain, the shear stress was chosen to analyze the influence of meniscal tear on the stress state in the innervated outer portion of the meniscus. However, specific shear stress thresholds that induce nociceptor activation are not reported in the current literature for the meniscus. Consequently, we postulate that a substantial relative increase in shear stress within the innervated region, compared to the physiological state in the intact meniscus, represents a key indicator of potential pain generation. In this paper, the Tresca equivalent stress was used to determine the shear stress state. The Tresca equivalent stress had been previously used in the correlation of meniscal stresses with the type of meniscal tears observed clinically (Pawar et al., 2024) and in the investigation of the effect of medial meniscus radial tears on the stress distribution (Wang et al., 2022).

The shear stresses at the nodes on the contour plots were averaged based on the values from neighboring elements (averaging threshold 100%) to minimize the potential numerical effect of stress concentration in a single finite element at the meniscal tear edge. The maximum shear stress and mean shear stress on the outer surface of the medial meniscus were used to assess changes in the stress state in the innervated part of the meniscus. The mean shear stress was determined from finite elements values from a region on the outer surface approximately 3 mm wide at the end of the tear. The finite element sets used to calculate the mean shear stress are presented in Fig. A in Supplementary Material for each meniscal tear model.

## Results

**FIGURE 3:**
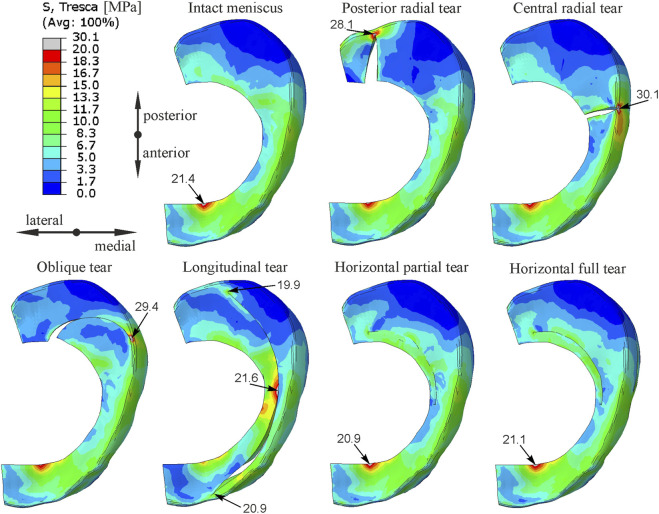
The contour plots of Tresca equivalent stress on the bottom surface of medial meniscus for the first peak of total force in the gait cycle.

The distribution of shear stress in the medial meniscus in different types of meniscal tears obtained from gait analysis is compared in Figure 3. Results were presented only for the first peak of total force corresponding to 25% of the stance phase, because at this time point the extreme stresses were achieved in the medial meniscus. The largest increase in the maximum shear stress relative to the intact meniscus model was observed for the radial tear of the body (40.6%) and for the oblique tear (37.4%). However, the greatest changes in the shear stress distribution were observed for the longitudinal tear model, in which the highest shear stress was obtained in the inner part of the middle body (Figure 3).

**FIGURE 4:**
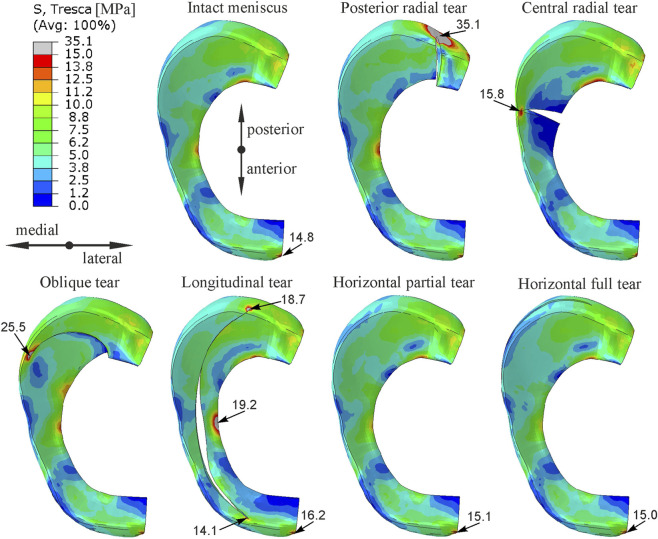
The contour plots of Tresca equivalent stress on the top surface of medial meniscus for the external tibial rotation combined with compression.

Figure 4 shows the contour plots of shear stress obtained for the external tibial torsion under load for all knee models. The contour plots are presented in Figure 4 on the top surface of the medial meniscus because higher stresses were observed there than on the bottom surface. The largest increases in maximum shear stress of 137% and 72% were observed for the posterior horn radial tear and oblique tear, respectively. In the longitudinal meniscal tear model the highest shear stress was obtained at the inner edge of the middle body, but increased stresses were also observed at both ends of the tear. In this model, a meniscal tear gap size increased by a maximum of 1.5 mm in its central part. No changes in stress distribution were observed in models with partial and full horizontal meniscal tear (Figures 3, 4).

The maximum and mean values of shear stress in the medial meniscus were compared in Table 1 for two loading scenarios and different types of meniscal tears. The highest maximum and mean shear stresses were obtained for the radial tear of the posterior horn. While, the largest increase in mean shear stress relative to the intact meniscus model was observed for the oblique tear. For the longitudinal tear, higher mean shear stresses were obtained at the posterior end of the tear and these are given in Table 1.

**TABLE 1:** Comparison of maximum and mean values of Tresca equivalent stress in the medial meniscus for different types of meniscal tears.

Model	Maximum shear stress [MPa]		Mean shear stress [MPa]		Increase in mean shear stress	
	1st peak of gait	Torsion + compression	1st peak of gait	Torsion + compression	1st peak of gait	Torsion + compression
Intact meniscus	21.4	14.8	​	​	​	​
Posterior radial tear	28.1	35.1	12.2	14.8	177%	75%
Central radial tear	30.1	15.8	8.1	6.9	59%	55%
Oblique tear	29.4	25.5	10.8	9.4	185%	187%
Longitudinal tear	21.6	19.2	6.1	8.0	178%	37%
Horizontal partial tear	20.9	15.1	6.9	4.2	3%	7%
Horizontal full tear	21.1	15.0	9.1	5.7	2%	26%

The values were reported for the first peak of total force in the gait cycle and external tibial torsion combined with compression. The mean values of shear stress were computed on the outer surface of the medial meniscus, see Fig. A in Supplementary Material. The increase in mean shear stress was calculated relative to the mean value determined for the same region in the intact meniscus model.

**FIGURE 5:**
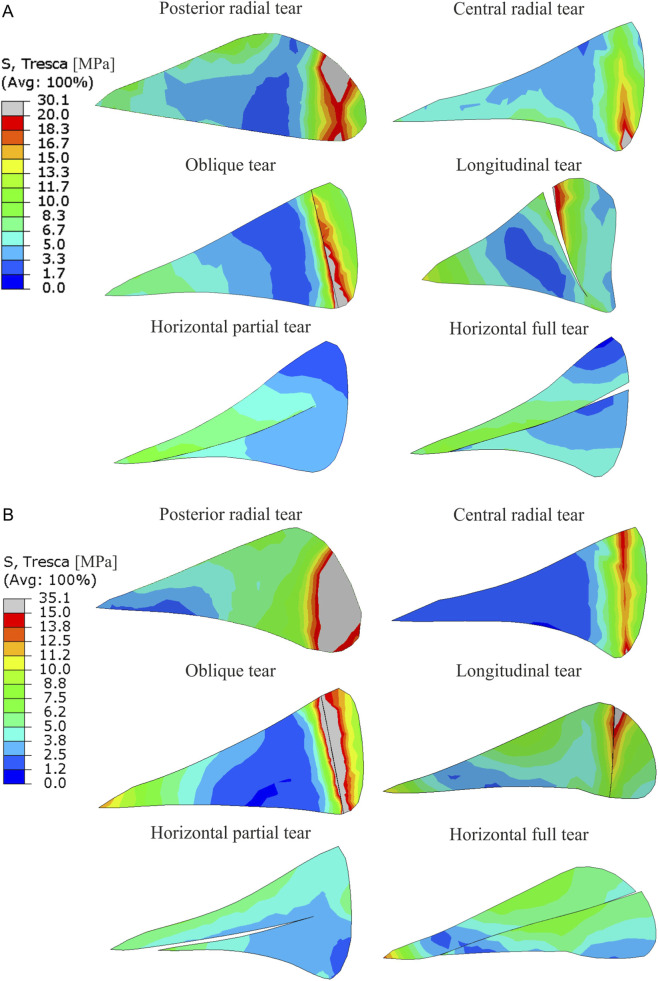
The contour plots of Tresca equivalent stress in the cross section through the medial meniscus tear for the first peak of total force in the gait cycle **(A)** and for the external tibial rotation combined with compression **(B)**.

The greatest increase in shear stress was observed at the end of the medial meniscus tear, therefore the stress distribution in the cross-section through the medial meniscus tear is compared in Figure 5. Contour plots of shear stress show that different types of vertical tears (radial, oblique, longitudinal) resulted in increased shear stresses in the innervated outer portion of the meniscus (Figure 5). The highest shear stresses were obtained in the models with posterior radial tear and oblique tear, see also Table 1. High shear stresses were also observed in the model with longitudinal meniscal tear and in the model with central radial tear at the top and bottom of the tear, respectively (Figure 5A). No increase in shear stress was observed for the horizontal tears of the medial meniscus (Figure 5).

Table 2 shows that the lowest contact force and total area in contact between the medial meniscus and tibial cartilage were obtained in the posterior radial tear model at the first peak of total force in the stance phase. The greatest decrease in contact force for the external tibial torsion under load was observed for the central radial tear. However, the decrease in contact force and contact area did not exceed 5% relative to the intact meniscus model in the torsion + compression analysis (Table 2).

**TABLE 2:** Comparison of total contact force and total area in contact between the medial meniscus and tibial cartilage determined for the first peak of total force in the gait cycle and for external tibial rotation combined with compression.

Model	Contact force [N]		Contact area [mm^2^]		Change in contact area	
	1st peak of gait	Torsion + compression	1st peak of gait	Torsion + compression	1st peak of gait	Torsion + compression
Intact meniscus	643.39	550.77	253.53	209.65	​	​
Posterior radial tear	592.18	536.20	221.93	205.91	−12.5%	−1.8%
Central radial tear	609.34	525.09	234.36	203.70	−7.6%	−2.8%
Oblique tear	592.65	533.31	223.53	202.69	−11.8%	−3.3%
Longitudinal tear	619.06	546.59	237.13	207.23	−6.5%	−1.2%
Horizontal partial tear	659.07	547.88	247.94	206.67	−2.2%	−1.4%
Horizontal full tear	650.74	544.08	237.24	200.18	−6.4%	−4.5%

## Discussion

In the current study, the influence of meniscal tear on the stress state in the innervated outer portion of the meniscus was analyzed in the four most common types of meniscal lesions. Particular attention was paid to the increase in shear stress, as excessive stress may activate the nociceptors and lead to meniscal pain. Analyses were performed for two loading scenarios using only one patient-specific FE model, because their aim was to qualitatively describe the changes in the stress distribution rather than to quantitatively evaluate the results, which depend on many factors, such as tear geometry, patient-specific geometry, material properties and other model parameters. The peak shear stresses at the end of the meniscal tear determined in this study are in good agreement with values of 14–30 MPa obtained during gait analysis for radial tears involving 83% of the width (Wang et al., 2022). Similar to study (Wang et al., 2022), there was a relatively small effect of meniscal tears on peak contact pressure at the medial tibial cartilage and therefore these results were not presented in this study. The posterior and central radial meniscal tears resulted in a 12.5% and 7.6% decrease in contact area for the first peak of the gait cycle (Table 2), respectively, which is consistent with the 10.6% decrease in contact area obtained for radial tear involving 90% of the width in the cadaveric study (Bedi et al., 2010).

In both models with partial and full horizontal tear of the medial meniscus, we did not observe any increase in shear stress in the innervated zone of the meniscus, which may indicate a non-mechanical etiology of pain in this type of injury. Previous biomechanical studies (Spencer Jones et al., 1996; Yim et al., 2013; Brown et al., 2016) have shown that the horizontal meniscal tear does not significantly alter the biomechanics of the knee joint. This can be explained by the fact that in the horizontal tear the continuity of collagen fibers and circumferential stresses is preserved (Brown et al., 2016). Arno et al. (2015) showed that a horizontal cleavage lesion of the posterior horn of the medial meniscus resulted in a 6% decrease in contact area, which is consistent with the 6.4% reduction in contact area obtained for this type of tear at the first peak of the gait cycle (Table 2). Although horizontal tears are most common in knee osteoarthritis, they also occur in patients without any clinical symptoms (Zanetti et al., 2003). Some authors have shown no difference in pain relief between arthroscopic partial meniscectomy of the meniscus with horizontal tear and non-operative treatment (Yim et al., 2013; Kamimura et al., 2019). On the other hand, other authors indicated good results of treatment involving repair of this type of meniscal tear (Pujol et al., 2013; Kamimura and Kimura, 2014). Taking into account the above results and the fact that the gap of full horizontal tear closes during loading (Figure 5), it can be hypothesized that the tension of the joint capsule due to the outflow of synovial fluid from the gap may result in the development of pain. In further studies, a biphasic knee model taking into account the joint capsule and synovial fluid could allow for the verification of this hypothesis.

The performed numerical simulations showed that radial and oblique tears involving up to 90% of the meniscus width caused an increase in shear stress in the innervated zone of the medial meniscus (Figures 3, 4). Although a closed fracture gap was observed in the oblique tear and a gap opening in radial tears, in both types of meniscal tears the most important stress components are the circumferential stresses and shear stresses in the meniscus plane. The circumferential tensile stresses can only be transmitted through the narrow outer part of the torn meniscus, which leads to excessive stresses and the potential occurrence of pain. The greatest increase in maximum shear stress in gait and external tibial torsion analyses was observed in the model with radial tear of meniscal body and in the model with radial tear of the posterior horn, respectively (Table 1). However, radial tear of the posterior horn had greater influence on the stress state in cross section of the medial meniscus (Figure 5). Consequently, the greatest risk of meniscal pain during knee joint loading occurs with this type of meniscal tear. Although no relationship between different types of meniscal tears and pain was observed in clinical studies (Campbell et al., 2014; MacFarlane et al., 2017), arthroscopic partial meniscectomy resulted in significant pain relief in patients with central radial tear and oblique tear in comparative study of treatment efficacy in different morphological types of radial and horizontal meniscal tears (Kamimura et al., 2019). For these types of meniscal tears, a local increase in shear stress at the end of the tear was observed in numerical simulations (Figures 3, 4). The result of clinical study (Kamimura et al., 2019) may suggest that partial meniscectomy can reduce the stress concentration at the end of crack in the case of central radial tear and oblique tear. Pain relief was not observed in patients with radial tear of the posterior horn (Kamimura et al., 2019) because partial meniscectomy was probably unable to reduce the increased stresses on the outer surface of the meniscus, which according to our results are greatest in this type of tear, see mean shear stress in Table 1. However, to better understand the effect of partial meniscectomy on different morphological types of meniscal tears, further numerical analyses for partial meniscectomy models are necessary.

A separate issue is the influence of the size of the meniscal tear on the development of clinical symptoms. Previous studies (Bedi et al., 2010; Wang et al., 2022) have shown that radial lesions involving up to 60% of the meniscus width had very limited influence on knee biomechanics. Considering innervation of the meniscus in the vascular outer third and that the increase in stress was observed mainly at the end of the tear, it can be hypothesized that small radial tears do not cause pain. Another issue is the occurrence of pain in patients with medial knee osteoarthritis (OA), in which increased innervation of the medial meniscus was observed (Ashraf et al., 2011). Consequently, shorter meniscal tears may cause pain in such patients, because the results of simulations showed that stress concentration occurs at the end of the meniscal tear.

**FIGURE 6:**
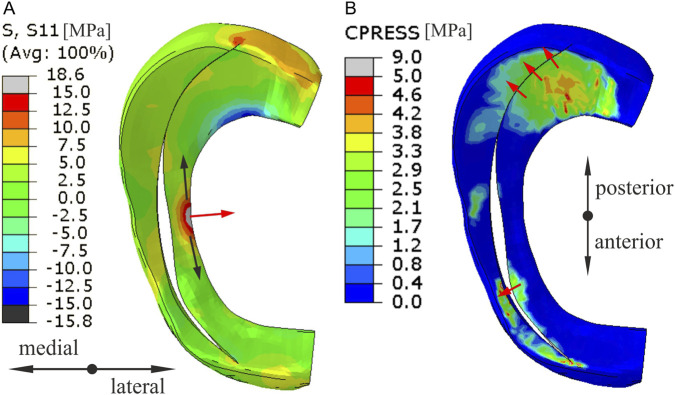
The contour plots of circumferential stress **(A)** and contact pressure **(B)** on the top surface of medial meniscus for the external tibial rotation combined with compression.

In the case of longitudinal tears, as in the case of other types of vertical tears, an increase in shear stress was observed at both ends of the tear. The stress concentration at the posterior end of the tear is caused by in-plane shear between the meniscal fragments and circumferential stresses (Figure 4). The shear stress concentration at the anterior end of the tear is caused by the opening of the meniscal tear gap. A larger tear gap was obtained at the central part of the meniscus in analysis of torsion with compression (Figure 4), but a larger gap at the anterior end of the tear was observed in the gait analysis (Figure 3). Moreover, the longitudinal tear caused significant changes in the shear stress distribution in the middle body of the medial meniscus (Figures 3, 4). Although the longitudinal tear does not disrupt the continuity of the circumferential fibers, it changes the way the medial meniscus transfer loads. The large tensile circumferential stresses at the inner edge of the meniscal body (Figure 6A) resulted in extension of the inner part of the medial meniscus and its inward displacement, which is not counteracted by contact stresses (Figure 6B). While, contact pressure acting on the inner part of the meniscus leads to closure of the tear gap in the posterior horn (Figure 6B). The inward displacement of the inner part of meniscus may result in meniscal instability and a bucket handle tear, and subsequent meniscus pain and knee locking. The longitudinal tear caused also a 10% increase in shear stress in the innervated anterior horn of the meniscus during external tibial rotation (Figure 4). In the case of a bucket handle tear, this increase may be much greater. Our results are consistent with clinical observations that inward displacement of the meniscus is necessary to induce pain in longitudinal tears (Corea et al., 1994), because in this type of tear the maximum and mean shear stresses in the innervated part of the meniscus were lower than in other types of vertical tears (Table 1).

This study has some limitations. In the FE model, the single-phase, hyperelastic Yeoh material model was used to describe the behavior of the articular cartilage, and an elastic, transversely isotropic material was used to model the menisci. A more detailed model, such as fibril-reinforced poroelastic (FRPE) material model, could describe the stress distribution more precisely but it is computationally demanding and difficult to implement. Moreover, Simkheada et al. (2023) showed that a simpler material model, such as an elastic orthotropic material, can also adequately model the meniscal response. Since an elastic model was used for cartilage and menisci, the phenomena of dissipation and stress relaxation were not considered. However, at gait frequency (approximately 2 Hz), relatively low energy dissipation and a small phase angle have been reported for the meniscus in previous studies (Morejon et al., 2022; 2023). Therefore, this simplification is expected to minimally overestimate peak stresses (Cognetti and Bedi, 2025). The flexion rotation and standardized loads obtained from the implant were used in the gait analysis. They may differ slightly from the real values for a healthy person. Only two load schemes were simulated in numerical analyses. Analysis of other activities, e.g., descending knee motion or squat, may provide additional information on the causes of meniscus pain and is therefore planned for further research. Another limitation of the study is the qualitative analysis of stress changes in the innervated part of the meniscus across different types of meniscal tears, due to the absence of defined stress thresholds that trigger nociceptor activation. The development of a micro-scale meniscus model to characterize the relationship between stress states at the tissue and cellular levels is planned for subsequent studies. Such a model would enable comparison of our findings with threshold values reported in the literature for other tissues (Dubin and Patapoutian, 2010; Delmas et al., 2011; Gong et al., 2022).

## Conclusion

Increased shear stresses in the innervated part of the meniscus, which may result in pain, were obtained in radial and oblique meniscal tears models in which the tear gap reaches the innervated part of the meniscus. Neither complete nor partial horizontal tears, in any of the assessed activities, were associated with changes in shear stress in the innervated zone of the meniscus. In case of longitudinal tear increase in shear stress was observed in the central part of the meniscus and at both ends of the tear. Additionally, instability of the inner part of the meniscus has been observed, particularly during external tibial rotation, which may cause increased stress on the innervated horns of the meniscus. As the calculations were performed for a single knee geometry and a linear transversely isotropic meniscus model, the results require further analysis and validation before clinical guidelines and recommendations can be formulated.

## Data Availability

The original contributions presented in the study are included in the article/Supplementary Material, further inquiries can be directed to the corresponding author.
