# Supplementary material for: Biomechanics of medial meniscus tears in the context of pain: a finite element analysis
Source: Front Bioeng Biotechnol. 2026 Jan 9;13:1693500. doi: 10.3389/fbioe.2025.1693500 (PMC12827652; doi:10.3389/fbioe.2025.1693500)
Supplement: Supplementary file 1 [file Supplementaryfile1.docx]

**Table A.** The stiffness and the initial strain for specific ligaments and ligament bundles.

| Ligament / ligament bundle | Stiffness (N) | Initial strain | Source |
| --- | --- | --- | --- |
| ACL - anteromedial | 5800 | 0.06 | (Blankevoort et al., 1991; Butler et al., 1992; Grzelak et al., 2012) |
| ACL – posterolateral | 3200 | 0.10 | (Blankevoort et al., 1991; Butler et al., 1992; Grzelak et al., 2012) |
| AIML | 750 | 0.00 | (Nelson and LaPrade, 2000; Guess and Razu, 2017) |
| ALL | 750 | -0.06 | (Kennedy et al., 2015; Drews et al., 2017) |
| LCL | 6000 | 0.05 | (Blankevoort et al., 1991; Yang et al., 2010) |
| MCL - anterior | 2400 | 0.03 | (Gardiner et al., 2001; Robinson et al., 2005) |
| MCL - middle | 2500 | 0.043 | (Gardiner et al., 2001; Robinson et al., 2005) |
| MCL - posterior | 2500 | 0.05 | (Gardiner et al., 2001; Robinson et al., 2005) |
| MCL - deep | 1300 | 0.00 | (Robinson et al., 2005; Smith et al., 2016) |
| PCL - anterolateral | 11400 | -0.16 | (Race and Amis, 1994; Moglo and Shirazi-Adl, 2003) |
| PCL - posteromedial | 2430 | -0.03 | (Blankevoort et al., 1991; Race and Amis, 1994) |
| POL | 1700 | 0.05 | (Wijdicks et al., 2010) |


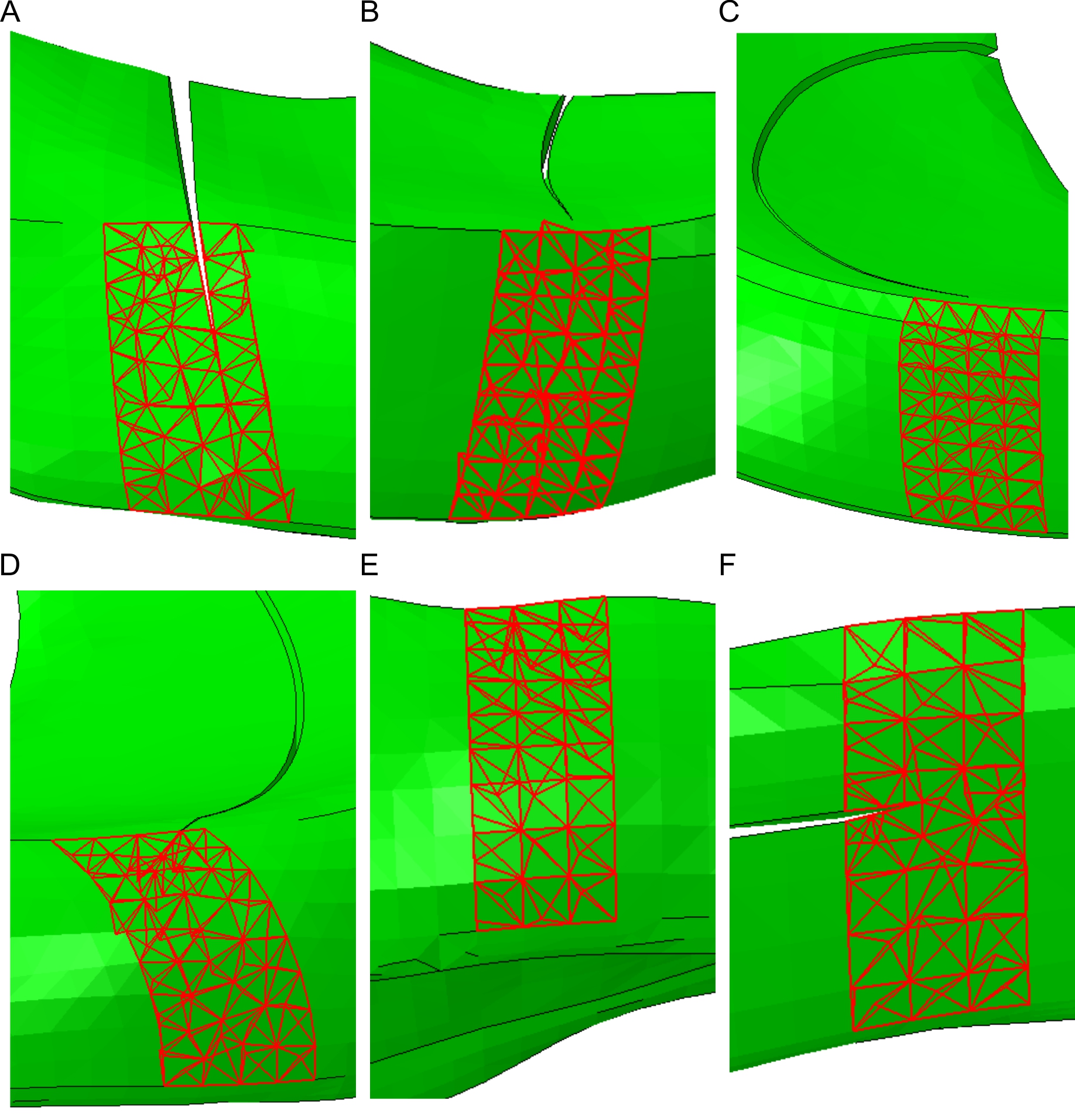


**Figure A**. The finite element sets used to calculate the mean shear stress on the outer surface of the medial meniscus for the following models: radial tear of the posterior horn (A), radial tear of the middle body (B), oblique tear (C), longitudinal tear (D), partial horizontal tear (E) and full horizontal tear (F).

**References**

Blankevoort, L., Kuiper, J. H., Huiskes, R., and Grootenboer, H. J. (1991). Articular contact in a three-dimensional model of the knee. *J. Biomech.* 24, 1019–1031. doi: 10.1016/0021-9290(91)90019-J

Butler, D. L., Guan, Y., Kay, M. D., Cummings, J. F., Feder, S. M., and Levy, M. S. (1992). Location-dependent variations in the material properties of the anterior cruciate ligament. *J. Biomech.* 25, 511–518. doi: 10.1016/0021-9290(92)90091-e

Drews, B. H., Kessler, O., Franz, W., Dürselen, L., and Freutel, M. (2017). Function and strain of the anterolateral ligament part I: biomechanical analysis. *Knee Surgery, Sport. Traumatol. Arthrosc.* 25, 1132–1139. doi: 10.1007/s00167-017-4472-3

Gardiner, J. C., Weiss, J. A., and Rosenberg, T. D. (2001). Strain in the human medial collateral ligament during valgus loading of the knee. *Clin. Orthop. Relat. Res.* Oct, 266–274. doi: 10.1097/00003086-200110000-00031

Grzelak, P., Podgorski, M. P., Stefanczyk, L., Krochmalski, M., and Domzalski, M. (2012). Hypertrophied cruciate ligament in high performance weightlifters observed in magnetic resonance imaging. *Int. Orthop.* 36, 1715–1719. doi: 10.1007/s00264-012-1528-3

Guess, T. M., and Razu, S. (2017). Loading of the medial meniscus in the ACL deficient knee: A multibody computational study. *Med. Eng. Phys.* 41, 26–34. doi: 10.1016/j.medengphy.2016.12.006

Kennedy, M. I., Claes, S., Fuso, F. A. F., Williams, B. T., Goldsmith, M. T., Turnbull, T. L., et al. (2015). The anterolateral ligament: An anatomic, radiographic, and biomechanical analysis. *Am. J. Sports Med.* 43, 1606–1615. doi: 10.1177/0363546515578253

Moglo, K. E., and Shirazi-Adl, A. (2003). On the coupling between anterior and posterior cruciate ligaments, and knee joint response under anterior femoral drawer in flexion: A finite element study. *Clin. Biomech.* 18, 751–759. doi: 10.1016/S0268-0033(03)00140-2

Nelson, E. W., and LaPrade, R. F. (2000). The Anterior Intermeniscal Ligament of the Knee: An Anatomic Study. *Am. J. Sports Med.* 28, 74–76. doi: 10.1177/03635465000280012401

Race, A., and Amis, A. A. (1994). The mechanical properties of the two bundles of the human posterior cruciate ligament. *J. Biomech.* 27, 13–24. doi: 10.1016/0021-9290(94)90028-0

Robinson, J. R., Bull, A. M. J., and Amis, A. A. (2005). Structural properties of the medial collateral ligament complex of the human knee. *J. Biomech.* 38, 1067–1074. doi: 10.1016/j.jbiomech.2004.05.034

Smith, C. R., Vignos, M. F., Lenhart, R. L., Kaiser, J., and Thelen, D. G. (2016). The influence of component alignment and ligament properties on tibiofemoral contact forces in total knee replacement. *J. Biomech. Eng.* 138, 021017–2. doi: 10.1115/1.4032464

Wijdicks, C. A., Ewart, D. T., Nuckley, D. J., Johansen, S., Engebretsen, L., and Laprade, R. F. (2010). Structural properties of the primary medial knee ligaments. *Am. J. Sports Med.* 38, 1638–1646. doi: 10.1177/0363546510363465

Yang, N. H., Canavan, P. K., and Nayeb-Hashemi, H. (2010). The Effect of the Frontal Plane Tibiofemoral Angle and Varus Knee Moment on the Contact Stress and Strain at the Knee Cartilage. *J. Appl. Biomech.* 26, 432–443.
